# Implementing prevention of seasonal affective disorder from patients’ and physicians’ perspectives – a qualitative study

**DOI:** 10.1186/s12888-018-1951-0

**Published:** 2018-11-26

**Authors:** Barbara Nussbaumer-Streit, Edda Pjrek, Christina Kien, Gerald Gartlehner, Lucie Bartova, Michaela-Elena Friedrich, Siegfried Kasper, Dietmar Winkler

**Affiliations:** 1 Department of Psychiatry and Psychotherapy, Medical University of Vienna, Währinger Gürtel 18-20, 1090 Vienna, Austria; 2 Department for Evidence-based Medicine and Clinical Epidemiology, Danube-University Krems, Dr.-Karl-Dorrek Strasse 30, 3500 Krems a.d, Donau, Austria; 3 RTI International, 3400 Cornwallis Rd, Research Triangle Park, NC USA

**Keywords:** Seasonal affective disorder, Winter depression, Prevention, Thematic analysis, Interviews

## Abstract

**Background:**

Seasonal affective disorder (SAD) is a seasonally recurrent type of major depression that has detrimental effects on patients’ lives during winter. Little is known about how it affects patients during summer and about patients’ and physicians’ perspectives on preventive SAD treatment. The aim of our study was to explore how SAD patients experience summers, what type of preventive treatment patients implement, which preventive treatment methods, if any, physicians recommend, and what factors facilitate or hinder implementation/recommendation of SAD prevention.

**Methods:**

We conducted 15 semi-structured interviews, ten with adult patients with a history of SAD and five with physicians. Transcripts were analyzed by two researchers using an inductive thematic analysis approach.

**Results:**

One group of patients was able to enjoy summer and ignore thoughts of the upcoming winter. The other group feared the impending depressive episode in winter, and this fear negatively impacted these patients’ well-being during the summer. Preventive treatment was a relevant issue for all patients, and all but one person implemented SAD prevention during summer. We identified six factors that influenced patient use of preventive treatment of SAD. Four factors occur on an individual level (*knowledge about disease and preventive treatment options, experience with treatment in acute phase, acceptability of intervention, willingness to take responsibility for oneself*), one on an interpersonal level (*social and work environment*), and one on a structural level (*healthcare system*). All psychiatrists recommended some kind of preventive intervention, most commonly, lifestyle changes. Four factors influenced psychiatrists in recommending prevention of SAD (*patient expectations, disease history and stability, risk/benefit ratio, lack of evidence*).

**Conclusions:**

Success in the implementation of SAD prevention does not solely depend on the willingness of the patients, but is also influenced by external factors. Raising awareness of SAD among general practitioners and low-level access to mental-health support could help patients find appropriate help sooner. To better guide the optimal treatment choice, comparative effectiveness research on treatments to prevent a new onset in patients with a history of SAD and clinical practice guidelines on SAD are needed.

## Background

Seasonal affective disorder (SAD) is a subtype of major depression that affects 2–8% of the total population in Europe [1–5]. It usually begins in fall/winter and remits in spring [6–8]. SAD is characterized by a high degree of persistence only resolving completely in one out of five patients after five to eleven years. In 22–42% of those affected, it persists with the seasonal pattern, and in 33–44% SAD turns into a non-seasonal major depression [9, 10].

Depressive episodes negatively impact patients’ social as well as working lives [11, 12]. In addition to depressive symptoms, most patients also experience hypersomnia, increased appetite often accompanied by weight gain, and extreme fatigue during winter months [13]. In summer, SAD patients are free of depressive symptoms. However, little is known whether or not the fear of upcoming depressive episodes impacts their well-being.

Light therapy is the first-choice treatment for acute SAD episodes and second-generation antidepressants are the second-choice treatment [14–16]. Other treatment options comprise agomelatine, melatonin, cognitive behavioral therapy, or lifestyle and diet changes [17–23]. The predictability of new depressive episodes in SAD patients provides a rationale for using these treatments preventively [24], beginning either in symptom-free summers or in fall when patients realize the first mild symptoms. A third approach to avoid the onset of a full-blown depression in the upcoming winter season is to continue acute treatment during summer.

Little guidance on the prevention of SAD exists. A German clinical practice guideline recommends starting light therapy in times of risk [25], while other guidelines do not provide specific recommendations for prevention of SAD [26, 27]. Also, the evidence on efficacy and safety of preventive treatment in SAD patients is limited. A systematic review demonstrated that the preventive use of the antidepressant bupropion extended release (XL) reduced the number of patients developing a new depressive episode in the next winter by 44% compared to placebo [placebo: 27% vs. bupropion XL; 15%] [28]. A randomized controlled trial on psychotherapy showed that cognitive behavioral therapy led to 27% recurrence of SAD episodes in the following winters compared to 46% with light therapy [29]. A pilot study in 46 SAD patients, however, showed that preventive mindfulness-based cognitive therapy did not prevent recurrence of SAD better than “treatment as usual” when administered in a symptom-free time [30]. No evidence on preventive efficacy of other antidepressants [28] or other types of psychotherapy exists [31]. Systematic reviews on preventive light therapy [32], on agomelatine, and melatonin [33] were not able to draw valid conclusions on efficacy and safety of these preventive treatments either. Nevertheless, preventive treatment is common in clinical practice. A recent survey in German-speaking countries demonstrated that 81 out of 100 interviewed hospitals recommend preventive interventions to their SAD patients, most frequently, lifestyle changes and antidepressants, followed by psychotherapy and light therapy [34].

When evidence from clinical studies is scarce and guidance from clinical practice guidelines is unavailable, treatment choice should be heavily based on patient preferences and the clinical expertise of physicians. To our knowledge, no study has yet qualitatively explored patients’ and physicians’ perspectives on prevention of SAD.

The aims of our study were to investigate how patients with a history of winter-type SAD experience summers, what type of preventive treatments they implement, if any, and what facilitates or hinders the implementation of preventive treatment in symptom-free periods. In addition, we strove to identify factors that influence physicians in prescribing preventive treatment to SAD patients.

## Methods

To understand what patients and physicians think about prevention of SAD and identify factors that influence the use and recommendation of interventions to prevent SAD, we interviewed both patients and physicians. The study was approved by and registered with the ethical review board of the Medical University of Vienna (EC No. 1586/2015). Throughout the manuscript, we adhere to the reporting guideline COREQ (COnsolidated criteria for REporting Qualitative research) for qualitative research [35].

### Participants

We recruited adult patients from the register of the SAD outpatient clinic at the Vienna General Hospital. We started the selection of patients with the most recent registration entries. We checked whether patients fulfilled inclusion criteria (18 years or older, history of SAD) and used purposive sampling. We selected interviewees from different age groups, genders, and durations of SAD in order to ensure a broad sample base. We aimed for an even distribution of gender to gain similar insight into female and male experiences.

We sent an invitation letter containing written patient information to potential interviewees. One week later, we contacted the patients by phone and asked if they were willing to participate in our study. We continued to invite patients to participate in the study until we reached data saturation (no new topics emerged from the interviews). Overall, ten patients participated in the study; twelve invited persons declined to participate (ten had no interest or time, and two lived abroad).

For interviews with physicians, we recruited three psychiatrists working in a hospital in Vienna and one psychiatrist working in private practice. The only inclusion criterion for psychiatrists was working experience with SAD patients. To gain a broader perspective on the topic, we selected both men and women psychiatrists with different ages and whose number of years of work experience with SAD patients varied. To confirm the findings of patient interviews which revealed that general practitioners play an important role in SAD patient decisions to seek help, we consulted a general practitioner. We identified the general practitioner and the private practice psychiatrist through our professional network. All five contacted physicians agreed to participate in our study and signed an informed consent.

### Interviews

An experienced qualitative researcher conducted semi-structured interviews. To ensure that patients were free of depressive symptoms at the time of questioning, interview sessions were scheduled between April and August 2016. Patients chose the interview setting (their home, office at the clinic, café, or via telephone). The interviews with the three psychiatrists took place in the psychiatrists’ offices at the clinic. The general practitioner and the private practice psychiatrist were each interviewed by phone. Before each interview, we received a signed informed consent from each participant. To ensure anonymity, we coded patient identities with numbers. Interviews lasted 20 to 40 min, irrespective of interview mode (face-to-face or phone). An audio recording was made of each interview and destroyed after study use. All interviews were transcribed verbatim. The researcher also recorded field notes after each interview. Because the researcher and patient met for the first time at the interview, and the researcher was not involved in the care of the patients, patients could be assured that their interviews would not affect their treatment in any way. The researcher used an interview guide developed by the study team in order to answer the a priori defined research question. The first questions addressed patients’ characteristics and patients’ SAD history; physicians were asked about their experience with SAD patients. The main part of the patients’ interviews focused on: how they experience summers; what preventive measurements they implement, if any; and what facilitates or hinders them from implementing preventive interventions. Physicians were asked what motivates and/or keeps them from recommending preventive treatment to SAD patients.

### Data analysis

We applied inductive thematic analysis as described by Braun & Clarke to identify, analyze, and report themes emerging from our data set – the interviews [36]. We chose thematic analysis because it focuses on the human experience similar to phenomenography [36]. First, we familiarized ourselves with the data and read and re-read the transcripts. Second, we assigned initial codes to the text. Third, we combined similar codes into subthemes and overarching themes. Fourth, we grouped themes into main categories. To facilitate the coding process, we used MAXQDA, Version 12.0 [37]. One experienced qualitative researcher performed the initial coding, grouping, and interpreting. As thematic analysis is an iterative process, the researcher discussed interim results (codes, subthemes, themes) and revised regularly with another researcher to corroborate findings.

## Results

We conducted 15 semi-structured interviews with ten patients and five physicians. We successfully created a diverse sample representing four female and six male SAD patients ranging between the ages of 20 to 60 years, with varying durations of the disease and differing living circumstances (see Table 1). The interviewed physicians represent two female and two male psychiatrists working in clinical or office-based settings as well as a male general practitioner, all with different degrees of experience in working with depressive patients (see Table 1).

**Table 1:** Characteristics of interviewees

Characteristics	Number of participants
Patients (*n* = 10)	
Gender	
Female	4
Male	6
Age	
20–29 years	2
30–39 years	3
40–49 years	3
50–60 years	2
Diagnosis of SAD	
≤ 5 years ago	3
6–10 years ago	3
11–20 years ago	2
> 20 years ago	2
Living arrea	
Suburban/rural area	4
Urban area	6
Living arrangement	
Lives with partner/family	6
Lives alone	4
Physicians (*n* = 5)	
Gender	
Female	2
Male	3
Age	
20–29 years	1
30–39 years	3
40–50 years	1
Working status	
Psychiatrist in a clinic	3
Office-based psychiatrist	1
General practitioner	1
Working with depressed patients	
Since ≤5 years	1
Since 6–15 years	1
Since 16–25 years	3

Abbreviations: *SAD* seasonal affective disorder, *n* number of participants

First, we describe how patients experienced summers and which preventive treatments patients and physicians mentioned implementing/recommending. Then, we present the six identified factors that influenced patients to use, and the four factors that influenced physicians to recommend preventive interventions.

### Patients’ experiences of summer

By the beginning of spring, patients experienced the end of a depressive episode, often as something quite sudden: *“In March, April, it [the depression] cleared away as if by magic. It was just gone”* (P_10). They perceived spring and summertime as positive contrasts to the depressive phase in fall/winter and felt clearly better during summer: *“I feel good now. I am productive. I can cope with stress. That is how I imagine my life to be. I am motivated and able to cope with my private and professional life”* (P_06).

However, while one group of patients managed to avoid any thought of a subsequent depressive episode during summer, the other group was worried about the onset of a new depressive episode throughout summer, and this negatively affected their well-being during symptom-free times, especially at the end of summer. One patient described the upcoming winter as a *“sword of Damocles”* (P_07). Another one said: *“You cannot enjoy the sun that much because you think, ah, it is mid-August. In one or two months it will start again”* (P_08)*.* In particular, those patients who had experienced more severe depressive episodes in previous winters already feared an upcoming episode in summer.

The severity of SAD differed strongly between interviewed patients. While some suffered from severe depression comprising suicidal thoughts and inability to participate actively in their social and professional life, others described their depressive episodes as mild. Despite different degrees of severity, all patients reported consequences to their professional and social lives. They felt a lack of drive and motivation. During depressive episodes, their performance at work dropped noticeably. Some were even unable to go to work at all. In addition, many interviewees had difficulties keeping up social contacts during winter. The concept of preventing the onset of a new depressive episode was relevant for all patients.

With the beginning of fall, some noticed signals like fatigue or lack of motivation as a sign that a new depressive episode was on the way: *“There are signals. I don’t want to go out anymore, don’t want to meet people, and I am really tired. […] Usually I read the newspaper every day. As soon as I realize I haven’t read the newspaper for three days, I know – these are the first signs – I have to be alert”* (P_05). For others the depressive episode came completely out of nowhere: *“I always have the feeling as if I slip into this from one day to another. It hits me with surprise”* (P_09).

### Preventive treatment mentioned by interviewees

All patients except for one implemented, and all psychiatrists recommended, some kind of preventive intervention. The most common was lifestyle changes such as spending time outside, physical activity, or a balanced diet (see Table 2). Half of the patients came up with the idea of preventive interventions by themselves; the other half received specific recommendations from their physicians.

**Table 2:** Preventive treatments mentioned in interviews (arranged by frequency)

Category	Interventions	Used/recommended by
Lifestyle changes	• Physical activity • Spending time outdoors • Structured lifestyle (e.g. appointments, hobbies) • Vacation in sunny regions • Balanced diet	Patients and physicians
Antidepressants	• Preventive use of antidepressants • Maintenance therapy with antidepressants	Patients and physicians
Light therapy	• Starting light therapy in fall before symptoms • Starting light therapy with first mild symptoms	Patients and physicians
Psychotherapy	• Continuation of therapy	Patients and physicians
Other interventions	• Vitamin D • St. John’s wort • Relaxation techniques (e.g. massages) • Alternative treatments (e.g. qi gong, osteopathy)	Patients

### Factors influencing whether or not patients implement prevention of SAD

We identified six factors that influenced patients in their use of preventive treatment of SAD. Four acted on an individual, one on an interpersonal, and one on a structural level (see Table 3).

**Table 3:** Factors influencing preventive behaviour in SAD patients

Level	Factor (Theme)	Sub-factor (Subtheme)
Individual	Knowledge about disease and preventive treatment options	• Patients’/Physicians’ knowledge about mechanisms of SAD • Patients’/Physicians’ knowledge about preventive treatment options
Individual	Experience with treatment in acute phase	• Patients’ experience with treatment in acute phases in terms of efficacy • Patients’ experience with side effects of treatment
Individual	Acceptability of intervention	• Perception of SAD as biologically/psychosocially caused • Attitude toward treatment options
Individual	Willingness to take responsibility for oneself	• Psychological strain • Time of disease experience • (Lack of) discipline
Interpersonal	Social and work environment	• Support from social network • Compatibility with daily routines
Structural	Healthcare system	• Access • Costs

Abbreviation: *SAD* seasonal affective disorder

#### Knowledge about SAD and preventive treatment options

For patients who understood the mechanisms of SAD and the high rate of recurrence, it was easier to implement preventive treatment because they perceived it as reasonable. For them it made sense to substitute natural light with artificial light therapy in times when daylight hours decreased. They also wished for more information provided by physicians: *“More information, more information – very important. In the end you are left alone with your problems […] more counseling by doctors would be nice*” (P_01).

However, patients often reported a lack of knowledge and awareness about SAD amongst their physicians, especially general practitioners. Upon noticing symptoms, most of the patients first consulted their general practitioners. However, none of the general practitioners consulted diagnosed SAD in the patients interviewed. Most of the general practitioners, in fact, refrained from referring the patients to a psychiatrist. Some interviewees felt that their general practitioners did not give serious consideration to their symptoms: “*If you go to a general practitioner in winter and tell him that you feel bad, he will say, ‘Well that is how it is in winter’”* (P_08). Only one patient visited a general practitioner who was aware of SAD and advised the patient to visit a psychiatrist to be formally diagnosed. The general practitioner interviewed in this study also questioned the existence of SAD.

Another important influencing factor was knowledge about treatment options. If patients and physicians did not know about an intervention, this, of course, hindered them from using/recommending it. Most patients learned about different interventions from their psychiatrists or psychotherapists. As a barrier for not implementing any preventive treatment, one patient mentioned he was not aware that this was even an option: “*I understood that you cannot do anything about it because you cannot change the seasons*” (P_07).

#### Experience with treatment in acute phases of depression

The type of experiences patients had with diverse treatments during acute depressive phases strongly influenced their attitudes toward using those treatments for prevention. Those who experienced light therapy or antidepressants as effective and well tolerated treatments in acute depressive phases were more willing to use them for prevention. One patient explained: *“I had the positive experience that drugs help me. And that is why I take them. As soon as I realize I’m not doing well, I’d better start right away”* (P_05). Negative experiences (e.g. no effect, side effects), however, lead to negative attitudes toward the use of preventive treatment. If a treatment did not show an effect in an acute phase, patients did not consider it for preventive treatment, as one statement of a patient talking about light therapy illustrates: *“If I had - I would have used it more intensively if I had noticed a change. I used it intensively in the beginning, but then I’ve used it less and less, after I did not notice any change”* (P_10).

#### Acceptability of intervention

Perception of SAD being caused mainly by biological or psychological factors influenced patients’ acceptability of treatment options. For patients with a biological perception of SAD, medication or light therapy seemed adequate treatment options while psychotherapy did not. *“So, I’m someone who tries to explain things chemically, that something is missing, synapses don’t work, and you can fix this with light therapy”* (P_06). To those who had a psychological understanding of SAD, psychotherapy seemed to be an essential treatment option, and light therapy or antidepressants were perceived as useful additional interventions but not sufficient when applied alone: *“I strongly believe that there is a connection between body and mind […] Light therapy only helps with symptoms […] I think my body will draw attention to something else with these symptoms”* (P_07).

What patients believe about interventions also strongly influenced their willingness to use those interventions for prevention. Many patients expressed a general negative attitude toward antidepressants. Patients tried to get along without drugs for as long as possible. Reasons for a negative attitude toward antidepressants were a general antipathy toward drugs, including drug usage in other circumstances, and in mood-changing substances specifically, as these two patients described: *“Before I take a pill for a headache, I try other things. I don’t want support from drugs. I look for alternatives”* (P_02). *“I am not a fan of drugs or pharmacological treatment because this only helps with symptoms not with the cause of the disease. That is why I try to change my lifestyle”* (P_03).

The psychiatrists confirmed this finding. Psychiatrists reported their patients’ dislike of antidepressants and preferences for non-pharmacological treatment: *“The majority of patients come to us and want light therapy. They have this expectation […] patients often simply don’t want drugs”* (D_03).

#### Willingness to take responsibility for oneself

A major driver for implementing preventive treatment in symptom-free times was the willingness to take responsibility for oneself, which was linked to the degree of psychological strain, long-lasting experience of disease, and discipline.

Patients who suffered a high degree of psychological strain from depressive episodes were more willing to start preventive treatment in symptom-free times. Due to the seasonal change of SAD, they knew how life could be without depressive symptoms and how burdensome it feels during fall/winter. The greater the psychological strain patients suffered during fall/winter, the more motivated they were to take preventive actions in symptom-free times: *“You know how bad you can feel in winter, and you learn from your experience. You know that you have to do something so that this doesn’t happen again. That is my motivation”* (P_09)*.* One interviewed physician agreed: *“This comes with high psychological strain. And they want to do something against these symptoms that they already know from the past winters”* (D_01).

Those patients who reported their depression as mild during winter were less motivated to undertake preventive treatment: *“I think I am at the beginning of my disease. I know how I feel when it gets worse. Well, I don’t have panic attacks, so I know that there are worse forms [of SAD]. That is not the case with me. Should it be worse this winter, I would need to think about it [preventive treatment]”* (P_02).

Patients experienced with SAD seemed to succeed more in implementing preventive interventions. Those patients who have experience the seasonal pattern over the years, tried different treatment approaches to find what works best for them. They have learned to recognize small changes in their mood and to interpret them as signals for the next onset of depression: *“In the meantime, I started to become more sensitive […] that was my learning over the last decades”* (P_05); *“I pay more attention to symptoms”* (P_10). Regimens in which patients start treatment before the onset of symptoms, require patient experience and patient recognition of early signals: *“When it became darker, I realized that symptoms were coming back, and then there was a point when it was nearly too late or at least it was critical, then I immediately went to the doctor and said, ‘Okay, I need medication’”* (P_06). Preventive treatment also requires support from a psychiatrist who can give patients the freedom to decide when they subjectively feel it is necessary to begin treatment.

The less experienced SAD patients were still optimistic that the upcoming winter would be depression-free: *“It got better in spring, and then I stopped treatment and thought, well, maybe next time it will not come back”* (P_10). During the summer they tended to ignore the impending depression: *“It doesn’t bother me at all in summer. Well, of course when it hits me in winter, it troubles me, but it’s not as if I already fear this phase in summer”* (P_04). *“In summer I just look toward the future. Everything is going to be brighter. No, there is nothing troubling me”* (P_02).

The implementation of preventive treatment during times when people feel well requires patient discipline. Patients often struggled to enjoy summer and to ignore every thought of an impending depressive episode: *“This is something you want to forget about. You don’t want to prepare for it because now, when you feel well, you want to enjoy it”* (P_07). Patients who were able to successfully implement preventive treatments like lifestyle changes credited their success to self-discipline: *“The older I got, the better it worked because I became more disciplined” (P_05). “That was my own internal process. I thought, I don’t want this anymore - to be lethargic all winter” (P_03).* Physicians also believed that successful implementation of preventive treatment during summer depended on patient discipline: *“Patients are often motivated to continue effective acute treatment to prevent the onset of new depressive phases, but then they don’t keep up with it”* (D_03)*.*

#### Social and work environment

Factors in the social and work environment, such as compatibility with daily routines, or social support, were perceived as facilitators, or if lacking, as barriers in the implementation of preventive treatments.

Compatibility with daily routines: some patients experienced preventive treatments as easy to integrate in their daily routines, e.g. light therapy: *“I could integrate it into my daily life. I have a desk job and sit in front of a computer. You only have to remind yourself to switch on the light”* (P_07)*. “It brought some structure into my life. I sat down, drank a cup of coffee, and looked into the light therapy device”* (P_06)*.* Others reported difficulties in implementing preventive treatments, e.g. due to their work or private duties. One patient who works nightshifts said: *“When I come home from a nightshift I would have to sit in front of a light therapy device, then I am awake again – that’s not working”* (P_05). *“For me it is not worth the effort” (P_04). “To be honest, the effort to sit in front of this lamp every day was time-consuming and a little bit difficult because of children and school and work”* (P_08)*.* Physicians reported similar difficulties from their patients: *“There are patients who have no desk job where they can install the lamp. I think this plays an important role”* (D_01)*.*

Social support and understanding from family and friends were perceived as facilitators, especially in helping patients to maintain a structured and active lifestyle and to seek help from psychiatrists and psychotherapists.

#### Healthcare system

We also identified influencing factors on a structural level – the healthcare system. Access to treatments and reimbursement of their costs worked as facilitators in implementing prevention.

Access to treatment: patients described difficulties in finding the “right” psychotherapist for them: *“Either you are lucky with your therapist or not […] if it fits – one cannot know in advance. You also don’t know if this will be better with the next therapist. And especially a patient suffering from depression will probably not think that the therapist might be the problem”* (P_01). Also finding a therapist within a reasonable timeframe was challenging: *“You have to wait up to a year and a half to get an appointment”* (P_05). Others criticized the lack of access to support for SAD patients: *“Why is there only one outpatient clinic for all the SAD patients in Austria?”* (P_08). Patients reported that it was easier to find a suitable therapist within a timely fashion if they visited doctors/therapists in private practices: *“My experience is that there is an extreme difference between the general health insurance system and the private system, especially in mental health”* (P_10).

Costs of treatment play a role, especially with light therapy and psychotherapy. The lack of coverage in health insurance plans for light therapy devices and psychotherapy treatments posed barriers for patients: *“If the insurance doesn’t cover treatment costs - that is a problem. I am the sole breadwinner at home. I have to feed two adults and a child. I definitely couldn’t afford weekly therapy sessions for 300€ a month” (P_01)*. Physicians also reported that the lack of health insurance coverage for certain treatments was problematic for many patients. On the other hand, when services were offered free of cost, insurance coverage acted as a facilitator: *“I take part in a health promotion program. […] I can get psychological counseling without paying out of my own pocket”* (P_05)*.*

### Factors influencing psychiatrists in recommending prevention of SAD

Four factors that influenced psychiatrists in recommending prevention of SAD were identified (see Table 4). Because the general practitioner did not recommend preventive SAD treatment; results are based on the interviews conducted with the four psychiatrists.

**Table 4:** Factors influencing physicians in recommending preventive treatment

Level	Factor
Patient	Patients’ expectations
Patient	Disease history and stability
Research	Risk/benefit ratio
Research	Lack of evidence

Psychiatrists recommended preventive treatment only to patients with a long and stable history of SAD. Only if the onset of a new depressive episode was very likely did psychiatrists advise patients to start with a treatment such as antidepressants or light therapy before symptoms occurred: *“In some patients, depressive episodes don’t return every season”* (D_02)*. “In my opinion, you have to consider if someone who might not develop a depressive episode should be treated without symptoms present”* (D_01).

Patient expectations influence what physicians recommend to them. If patients are unwilling to take antidepressants or go to psychotherapy, doctors will not recommend these treatment options, even if such options would be effective in prevention or in acute phases of depression. Sometimes patients demand specific treatments and are not open to other possibilities their physicians might suggest: *“The majority of people come to us and demand light therapy. If that is what they expect, it is hard to tell them that antidepressants are a good treatment option”* (D_03).

When considering preventive treatment, physicians weigh risks and benefits. Because SAD preventive treatments are initiated during symptom-free times and a physician can never be certain that a depressive episode will indeed develop, SAD preventive treatments present a particular challenge and may expose patients to unnecessary risks. Light therapy is preferred over antidepressants because the risk for potential side effects might be smaller compared to antidepressants.

The main barrier in recommending preventive treatments, such as melatonin or vitamin D, was a lack of evidence: *“It looks good, helps with sleeping problems, but as with antidepressants – I prefer to wait for study results”* (D_04)*.* Psychiatrists extrapolated from acute treatment settings to preventive treatment but wished for valid evidence about efficacy and safety of preventive treatment.

## Discussion

To the best of our knowledge, this is the first qualitative study to explore patients’ and physicians’ perspectives on prevention of SAD and to identify factors that facilitate or hinder its implementation. By using thematic analysis we grouped the insights of the vast amount of information into factors that influence SAD prevention on an intrapersonal, interpersonal, and environmental level. This is in line with the ecological model of health behavior that argues that an individual’s health behavior is not solely affected by internal factors but also by other people and context factors [38]. The typical purpose of qualitative research is to gain an understanding of complex situations and real-world problems, especially, in areas which lack research efforts like patients’ and physicians’ perspective on SAD prevention [35].

Our study was the first to formally assess that preventing the onset of a new depressive episode in winter was a relevant issue for all SAD patients, whether the fear of the next upcoming depressive episode negatively impacted their wellbeing in summer, or not. This is in accordance with findings from a Swedish study exploring patients’ experiences with SAD in general [12], and underlines the need for preventive treatment. This need was also demonstrated in a recent survey that showed that preventive treatment is recommended by more than 80% of psychiatric hospitals in German-speaking countries [34].

Changing lifestyle, taking antidepressants, or starting light therapy and psychotherapy before the onset of depressive symptoms were preventive treatments implemented by the interviewed patients. This coincides with what psychiatrists reported as recommending most often for SAD prevention [34]. In our interviews, psychiatrists also reported a lack of evidence on prevention of SAD leading to high uncertainty and a need for comparative effectiveness studies on preventive treatment options for SAD. Consequently, psychiatrists relied more on patients’ preferences and values, indicating good shared decision-making. The identified barriers and facilitators on the individual level can support psychiatrists in the counselling process of their patients to find an individual suitable preventative treatment.

One major barrier patients encountered when searching for help, was that general practitioners did not recognize SAD symptoms. Consequently, SAD patients often remain mis- or underdiagnosed and continued to suffer from symptoms and functional disability. This is especially alarming considering that 60% of depression-related treatment is provided by general practitioners [39]. To ensure that patients receive the immediate care they need, general practitioners have to be aware of the symptoms, mechanisms, treatment and preventive options of SAD. In particular, patients who are lacking experience with SAD and have not learned to master their disease need support from physicians and therapists. Raising general awareness of SAD is important so those who experience it can recognize their symptoms and receive the necessary support in their social and working environment.

The inability of patients to integrate preventive treatments into their everyday working and social lives highlights the need for a broad range of treatment options that can better meet patient needs. On a structural level, a lack of access to treatments and coverage of costs pose barriers to treatment. Low-level access to and financial support for treatment of mental health problems is needed, especially for light therapy, which is recommended for clinical practice [25].

One limitation of our study is that we did not formally evaluate if patients still fulfilled diagnostic criteria for SAD, but relied on diagnoses documented in patient charts and on patient self-reports. However, since we were interested in experiences of SAD patients, it was essential that they had a history of SAD, which was confirmed by documented diagnoses. Second, we interviewed only a few psychiatrists, three of whom worked at the same institution. However, because the institution is specialized in the treatment of SAD patients, the psychiatrists selected have extensive experience with SAD patients. Although the initial research plan did not foresee an interview with a general practitioner, this component was added due to the amount of patients who reported their general practitioners as key persons in their search for help with SAD. Therefore, the interview guide was slightly adapted for the general practitioner interview. Third, patients were interviewed about past experiences and results could therefore be influenced by recall bias. Fourth, the method of interview differed for some interviewees. Two patients and two physicians preferred to be interviewed via phone due to logistical reasons. We cannot rule out that this affected the willingness to share personal experiences. However, the duration of the interviews via phone was comparable to the face-to-face interviews, the interviews followed the guiding questions, and no one listened to the conversation, ensuring a trustworthy atmosphere.

A special strength of our study is, that the researcher who conducted all patient interviews was in no way involved in the care provided to the patients and patients could be ensured that interview responses would not affect their treatment. This in turn, contributed to an atmosphere of trust and openness.

## Conclusions

Raising awareness of SAD, its symptoms, and treatment options among general practitioners and in society is essential to help patients find appropriate support sooner.

Since prevention begins when patients still feel well, it costs them quite an effort to implement preventive interventions. Considering the high probability of the onset of a new depressive episode, it is important to enable SAD patients to implement prevention. Therefore, it is advisable to provide a broad range of treatment options that fit the practical needs of patients and allow them to integrate an intervention into their daily routines. Reimbursement of treatment costs, low-level access and short waiting times for psychotherapy (CBT) would be beneficial. To better guide the optimal treatment choice, comparative effectiveness research on treatments to prevent a new onset in patients with a history of SAD and clinical practice guidelines on SAD are needed.

## Abbreviations

D: Doctor
n: Number of interviewees
P: Patient
SAD: Seasonal affective disorder
